# The Choice of Candidates in Survival Markers Based on Coordinated Gene Expression in Renal Cancer

**DOI:** 10.3389/fonc.2021.615787

**Published:** 2021-05-11

**Authors:** Natalya Apanovich, Pavel Apanovich, Danzan Mansorunov, Anna Kuzevanova, Vsevolod Matveev, Alexander Karpukhin

**Affiliations:** ^1^ Laboratory of Molecular Genetics of Complex Inherited Diseases, Research Centre for Medical Genetics, Moscow, Russia; ^2^ Department of Oncourology, Federal State Budgetary Institution “N.N. Blokhin National Medical Research Center of Oncology” of the Ministry of Health of the Russian Federation, Moscow, Russia

**Keywords:** renal cancer, gene expression, correlation, markers, survival

## Abstract

We aimed to identify and investigate genes that are essential for the development of clear cell renal cell carcinoma (ccRCC) and sought to shed light on the mechanisms of its progression and create prognostic markers for the disease. We used real-time PCR to study the expression of 20 genes that were preliminarily selected based on their differential expression in ccRCC, in 68 paired tumor/normal samples. Upon ccRCC progression, seven genes that showed an initial increase in expression showed decreased expression. The genes whose expression levels did not significantly change during progression were associated mainly with metabolic and inflammatory processes. The first group included *CA9*, *NDUFA4L2*, *EGLN3*, *BHLHE41*, *VWF*, *IGFBP3*, and *ANGPTL4*, whose expression levels were coordinately decreased during tumor progression. This expression coordination and gene function is related to the needs of tumor development at different stages. Specifically, the high correlation coefficient of *EGLN3* and *NDUFA4L2* expression may indicate the importance of the coordinated regulation of glycolysis and mitochondrial metabolism. A panel of *CA9*, *EGLN3*, *BHLHE41*, and *VWF* enabled the prediction of survival for more than 3.5 years in patients with ccRCC, with a probability close to 90%. Therefore, a coordinated change in the expression of a gene group during ccRCC progression was detected, and a new panel of markers for individual survival prognosis was identified.

## Introduction

Clear cell renal cell carcinoma (ccRCC) is the most common type of kidney neoplasm. It accounts for 80–90% of kidney cancer worldwide. ccRCC is characterized by high malignancy and results in the highest mortality rate among the genitourinary system cancers (1).

Despite the annual increase in the incidence of kidney cancer, the development of early detection technologies has reduced the mortality rate due to this disease in recent decades (2). However, the outcome in patients after nephrectomy varies significantly, and in patients who experience relapse or tumor metastasis, the survival rates are reduced to less than 20–30% of the total 5-year survival rate (3).

Information that enables medical professionals to predict the course of the disease is extremely important for selecting clinical approach and improving the effectiveness of treatment. The existing and applied prognostic systems that are based on histological and clinical parameters are not accurate enough to reliably detect risk (4–8). As a result, there is a need to develop new forecasting methods and biomarkers for detecting ccRCC.

In recent years, there has been an active search for molecular genetic markers that are differentially expressed in tumor and normal tissues and can be used in the early diagnosis and prognosis assessment of the disease. This information may help determine the degree of radical surgery required and the choice of drugs to be administered for effective treatment (9–11). However, predictive markers for kidney cancer that are currently recognized and used in practice are not yet available (1). The development and practical application of such biomarkers will significantly increase the accuracy of the prognosis, and consequently, improve the quality of treatment. The development of an effective system of prognostic markers will enable individualized treatment and contribute to the accurate prognosis of survival in individual patients.

The identification of genes whose expression significantly contributes to ccRCC progression and clarification their function during tumor progression may enable the choose effective biomarkers. Additionally, the study of such genes may contribute to the understanding of the mechanisms underlying ccRCC development. Therefore, it is essential to understand the coordination of genes that play a role in the development of ccRCC, which has not been studied sufficiently.

We aimed to identify genes whose expression significantly contributes to ccRCC progression, and based on these findings, to delineate prognostic markers. Of the 20 differentially expressed genes identified, seven genes coordinately demonstrated a reduction in the initial increase in expression resulting from ccRCC progression. Based on their coordinated expression, it was possible to conclude the functional features of these genes during ccRCC development, and we identified the relationship between these genes and patient survival. Further, we created a panel of four candidate-gene markers, whose expression was found to predict individual survival. The identification of these candidate-gene markers is an important step in the development of a prognostic system.

## Materials and Methods

### Patient Samples and Their Classification

Material for research and the identification of clinical characteristics were obtained at the Federal State Budgetary Institution «N.N. Blokhin National Medical Research Center of Oncology» оf the Ministry of Health of the Russian Federation. All subjects gave informed consent for inclusion in the study before participation. The study was conducted in accordance with the Declaration of Helsinki, and the research protocol was approved by the Ethics Committee of the Federal State Budgetary Institution “Research Centre for Medical Genetics” (approval number 2017-4/2). The sample consisted of Russian patients who were selected based on the primary ccRCC that was detected without any prior

Sixty-eight paired samples of tumor (ccRCC) and morphologically normal kidney tissues that were taken from the same organ were examined. The samples were freshly frozen surgical material. The normal kidney tissue was used as a control.

Histological data for the samples under study were obtained from the Department of Pathological Anatomy of Human Tumors, Federal State Budgetary Institution «N.N. Blokhin National Medical Research Center of Oncology» оf the Ministry of Health of the Russian Federation. The average age of the patients was 55 years (ranging from 19 to 77 years).

The samples were classified using the TNM classification from clinical stages I–IV. There were 15 (22.1%) stage I, 7 (10.3%) stage II, 17 (25%) stage III, and 29 (42.6%) stage IV samples. Because of the small number of stage II samples and the similarity of their clinical characteristics to stage I samples, the two sets of data were combined: stage I + II = stage I/II = 22 (32.3%). To analyze the relationship between the expression levels of these genes and survival outcomes, we studied the clinical survival data of 38 patients. Additionally, two groups of patients were identified based on life expectancy; patients who lived more than 3.5 years (23 samples) and those who lived less than 3.5 years (15 samples) from the time of diagnosis. The survival-tracking period was up to 8.5 years. More detailed information on sample characteristics is given in **Table 1**.

**Table 1:** Patient characteristics.

Patient characteristics	Age at diagnosis (years)	Gender	Demography: Region and Etnicity	Histology	T	N	M	Grade
Parameters—Number of cases	Median—55	Men—44 (63%)	Сentral region of Russia—100%	ccRCC—70 (100%)	1—11 (16%)	0—57 (81%)	0—46 (66%)	1—9 (13%)
	Range—19–77	Women—26(37%)	Russians—59 (84%)		1a—2 (3%)	1—4 (6%)	1—24 (34%)	2—34 (49%)
			Others—11 (16%)		1b—6 (9%)	2—7 (10%)		3—20 (29%)
					2—14 (20%)	X—2 (3%)		4—1 (1%)
					3—1 (1%)			X—6 (9%)
					3a -16 (23%)			
					3b—14 (20%)			
					3c—2 (3%)			
					4—2 (3%)			
					X—2 (3%)			

### Gene Expression Analyses

Gene selection was performed based on methods described in our previous research (12). The analysis of microarray expression databases enabled the selection of the 200 most highly and frequently expressed genes in ccRCC tumors. The expression of these genes was studied using the real-time polymerase chain reaction (RT-PCR) method on 20 paired samples of tumor/normal tissue, from which 20 genes with the highest and most frequent expression in ccRCC were selected. Expression of the following 20 genes were studied in 68 ccRCC tumor samples: *CA9*, *NDUFA4L2*, *HIG2*, *INHBB*, *IGFBP3*, *ANGPTL4*, *EGLN3*, *VWF*, *TYROBP*, *BHLHE41*, *STC2*, *MMP9*, *CXCR4*, *NNMT*, *CSF1R*, *FN1*, *PFKP*, *SLC16A3*, *C1QA*, and *CD36*. Genes from each ccRCC tumor sample were paired with and compared to those from a sample of normal tissue from the same kidney.

The commercial RNeasy Mini Kit (Qiagen, Maryland, USA) was used to isolate total RNA from tumor and normal kidney tissue samples. The presence and intensity of 28S/18S rRNA bands of the total RNA were checked *via* electrophoresis on a 1.8% agarose gel using the Bio-Rad Subcell horizontal electrophoresis chamber (Bio-Rad, California, USA) and gel imaging with the GelDoc XR+ imaging and gel documentation system (Bio-Rad, California, USA). The quantity and quality of RNA was evaluated using the Nanodrop-ND 1000 UV–Vis Spectrophotometer (Thermo Fisher, Massachusetts, USA). The RNA was considered to be of acceptable quality if the two bands corresponding to the 28S and 18S rRNA had an intensity ratio of ~2:1, and the A260/A280 ratio was 1.8–2.1. The ImProm-II ™ Reverse Transcription System kit (Promega, Madison, USA) was used for the reverse transcription reaction. RT-PCR was performed using the TaqMan^®^ Gene Expression Master Mix (2-fold universal reaction mixture for studying the level of gene expression by RT-PCR), and the TaqMan^®^ Gene Expression Assay (reaction mixture containing primers and fluorescently labeled oligonucleotide) (Applied Biosystems, California, USA) for each gene. RT-PCR was performed in triplicate for each gene along with a no-template negative control. The *GAPDH* gene was used as an endogenous control (13). The relative level of mRNA expression of each gene was calculated in tumor tissues relative to normal kidney tissue using Step One Software (Applied Biosystems, California, USA) for the ΔΔCt (RQ) method (14).

### Survival Analysis and Statistics

The Receiver Operator Characteristic (ROC) analysis was performed using the MedCalc software (MedCalc software Ltd, Ostend, Belgium). The cutoff values were used to analyze the relationship between the level of mRNA expression and the survival of patients with ccRCC by assessing the significance of the differences in the survival curves using the Kaplan–Meier method. This method is often used to analyze the relationship between survival and the factors influencing it, is widely used, and reliable (15, 16). Survival curves were constructed by classifying patients based on the level of gene expression that was above or below the cutoff value as determined by the ROC analysis. The statistical significance of the differences in the survival curves was assessed using the log-rank test.

An online calculator (https://www.medcalc.org/calc/diagnostic_test.php) was used to calculate the sensitivity and specificity of a panel of markers and the odds ratio (OR) and relative risk (RR). The OR and RR values were used to quantify the relationship between the expression levels of the studied genes and the survival outcomes. Statistical analysis of the data was performed using Statistica 10.0 (StatSoft Inc., Tulsa, USA). The differences in expression levels were assessed using the Mann–Whitney U test. The significance of the association of the expression level of these genes with the TNM stage of ccRCC and a life span of more and less than 3.5 years was assessed using the Fisher’s exact test The Cox proportional hazards model and log-rank test were used to identify and assess the relationship between expression levels and patient survival. The significance level was set at *p <*0.05. To identify the presence or absence of coordination of gene expression changes during tumor development, Spearman’s correlation analysis was performed. The result was considered significant at *p* <0.01.* A* gene enrichment analysis was performed using the GO database (http://geneontology.org) to understand the processes in the ccRCC tumors.

## Results

The expression levels of the 20 genes under investigation were determined in 68-paired kidney tissue samples (tumor/normal). The stage I/II ccRCC samples showed an increase in the expression of *CA9*, *NDUFA4L2*, *HIG2*, *INHBB*, *IGFBP3*, *ANGPTL4*, *EGLN3*, *VWF*, *TYROBP*, *BHLHE41*, *STC2*, *NNMT*, *SLC16A3*, *C1QA*, and *CD36* relative to normal kidney tissue. The increase in expression of the *CXCR4*, *PFKP*, and *FN1* genes was less than two-fold. The *CSF1R* gene in stage I/II samples was expressed at a low level of difference from the normal tissue. No increase in the expression of *MMP9* was observed in the stage I/II samples, although its expression was increased in stage III samples (**Table 2**).

**Table 2:** Gene expression levels and the significance of their differences at different stages of ccRCC development.

Gene	Me (I + II)	Me (III)	Me (IV)	P =, U-test*
*NDUFA4L2*	29.4	26.9	1.1	0.003
*CA9*	26.9	21.0	1.2	<0.001
*HIG2*	10.8	6.4	9.5	0.715
*ANGPTL4*	8.7	9.6	1.1	<0.001
*EGLN3*	9.2	5.0	0.8	<0.001
*INHBB*	4.5	3.5	5.5	0.964
*TYROBP*	4.2	1.3	2.1	0.832
*NNMT*	4.1	3.2	2.0	0.523
*BHLHE41*	3.7	2.3	1.2	0.005
*STC2*	3.5	7.8	0.4	0.138
*SLC16A3*	3.1	3.7	4.6	0.477
*IGFBP3*	2.5	2.9	1.0	0.027
*VWF*	2.4	1.7	0.7	0.004
*CD36*	2.3	1.0	2.1	0.551
*C1QA*	2.0	0.8	1.0	0.203
*CXCR4*	1.8	0.8	0.9	0.213
*PFKP*	1.5	1.4	0.6	0.091
*FN1*	1.3	1.3	1.2	0.45
*MMP9*	1.1	2.3	0.7	0.325
*CSF1R*	0.8	0.9	1.0	0.483

*Comparison of expression levels at stages I + II and IV.

The expression of seven genes was significantly lower in stage IV relative to stages I/II; i.e., *CA9*, *NDUFA4L2*, *EGLN3*, *BHLHE41*, *VWF*, *IGFBP3*, and *ANGPTL4* (p = 0.001–0.027) (**Table 2**). The gene expression levels at the 3rd stage occupies an intermediate position between 1 and 4th stages, but it does not differ statistically significant. No significant changes were observed in the expression levels of the remaining genes.

**Figure 1:**
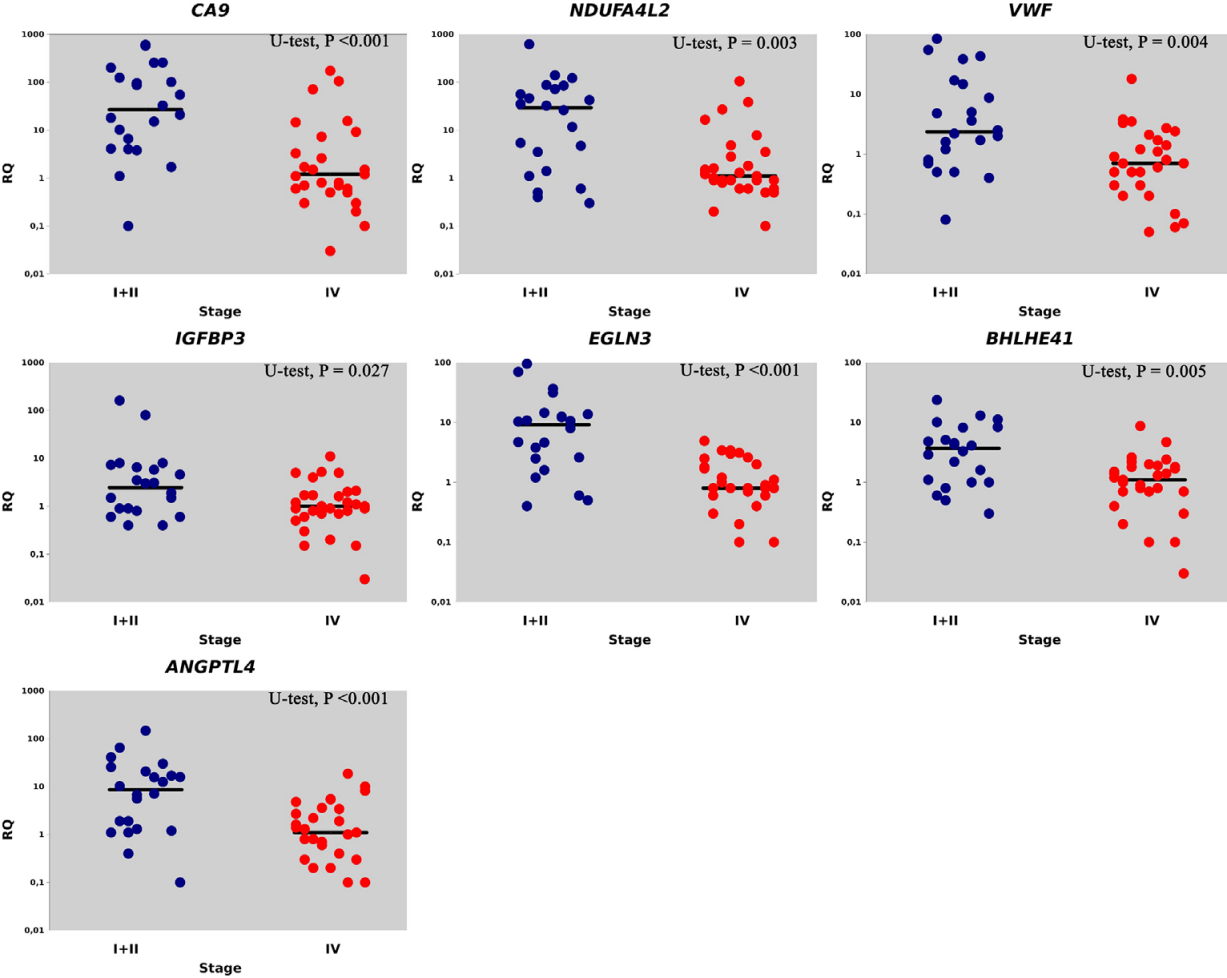
Relative gene expression (RQ) in I + II (●) and IV stages (●). Gene expression values are presented on a logarithmic scale. The line marks the median.

**Figure 1** shows the values of the gene expression levels and the median statistically significant difference between the expression levels of genes from stages I/II ccRCC as compared to stage IV.

Furthermore, we assessed the relationship between the studied gene expression and developmental stages of ccRCC. ROC analysis revealed the cutoff values for expression levels of stage I/II and stage IV ccRCC that exhibited the best sensitivity and specificity. For each gene in stages I/II and IV, the frequency of expression that was above and below these cutoff values was determined. Based on the results from the ROC analysis and Fisher’s exact test, there was a significant association (p = 0.001–0.034) of the expression level of these seven genes with the TNM stage (**Table 3**). The significance of the difference in the expression frequencies of the studied genes that were above and below the cutoff value for stages I/II and IV remained after applying the false discovery rate (FDR) correction for multiple comparisons for both the statistical methods.

**Table 3:** Results of ROC analysis of differences in gene expression at stages I, II and IV of ccRCC, frequency of gene expression levels in tumors relative to the cutoff value, and association of expression with tumor stages based on the Fisher exact test.

Gene	ROC analysis, p =	Cutoff value	Frequency of expression higher/lower from the cutoff value in the I+II stages	Frequency of expression higher/lower from the cutoff value in the IV stages	Fisher exact two-tailed, p=
*CA9*	<0.001	≤3.3	19/3	7/22	<0.001
*NDUFA4L2*	0.002	≤2.8	16/6	7/22	<0.001
*EGLN3*	<0.001	≤3.4	15/7	1/28	<0.001
*BHLHE41*	0.001	≤2.6	13/9	2/27	<0.001
*VWF*	<0.001	≤1.4	15/7	8/21	0.005
*IGFBP3*	0.034	≤2.1	11/11	5/24	0.017
*ANGPTL4*	<0.001	≤5.5	14/8	3/23	<0.001

Consequently, the expression of *CA9*, *NDUFA4L2*, *EGLN3*, *BHLHE41*, *VWF*, *IGFBP3*, and *ANGPTL4* is associated with ccRCC tumor progression and decreases at stage 4 after initial growth relative to normal tissues. The expression of other differentially expressed genes did not exhibit statistically significant changes during the progression of ccRCC.

**Figure 2:**
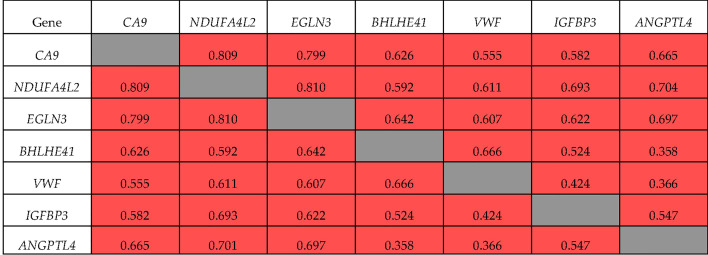
Correlation of gene expression during tumor progression. ■ significant correlation.

Spearman’s correlation analysis showed that all seven genes had mutually correlated expression levels (*p <*0.01), and formed a co-expression cluster (**Figure 2**). The expression of *ANGPTL4* with *BHLHE41* and *VWF* (R = 0.358 and 0.366, respectively) demonstrated the lowest correlation coefficients. The expression of *NDUFA4L2*, *EGLN3*, and *CA9* had the highest correlation coefficient (R = 0.799–0.810). The expression of *ANGPTL4* and *IGFBP3* was correlated with these genes (R = 0.582–0.704).

**Figure 3:**
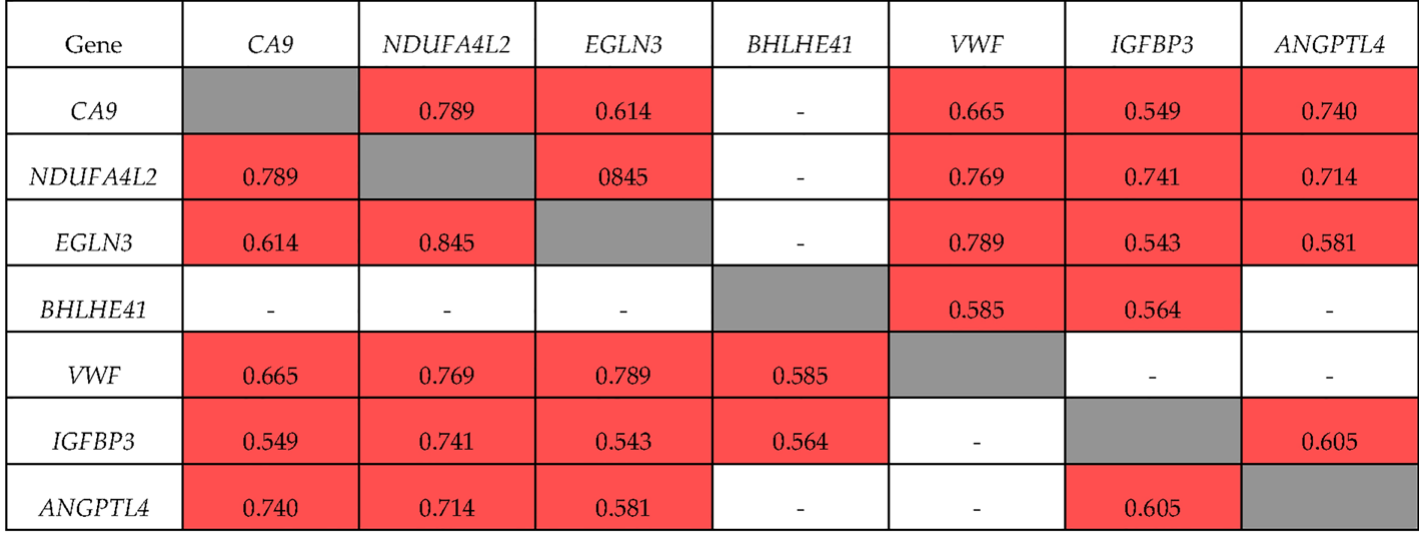
Correlation of gene expression at stage I + II ccRCC. ■ significant correlation; □ there is no significant correlation.

The correlations of these genes in ccRCC Stage I/II were somewhat different (**Figure 3**). Specifically, the expression of *BHLHE41* was correlated with the expression of *VWF* and *IGFBP3* alone, and the expression of *ANGPTL4* at Stage I/II did not correlate with that of *VWF* and *BHLHE41*. There was no correlation between the expression of *IGFBP3* and *VWF*. For *NDUFA4L2*, *EGLN3*, and *CA9* expression, the values of the correlation coefficients were similar to those observed during tumor progression, i.e., R = 0.845 and 0.789, respectively.

The processes in tumors of ccRCC that were enriched using the studied genes are presented in **Table 4**. Genes, whose expression was associated with the progression of ccRCC, were studied for a possible relationship with progression-free survival.

**Table 4:** The GO processes involving the genes under study.

Accession	Biological process (GO)	Genes	raw P value	FDR
GO:0071456	cellular response tohypoxia	*CA9, HIG2, EGLN3*,*STC2*	3.31E−05	4.41E−02
GO:0006950	response to stress	*CA9, HIG2, INHBB,ANGPTL4, EGLN3, VWF, TYROBP, STC2, MMP9, CXCR4, CSF1R, FN1, C1QA, CD36*	3.50E−07	5.58E−03
GO:0001666	response to hypoxia	*CA9, HIG2, EGLN3, STC2, ANGPTL4, CXCR4*	7.12E−07	5.68E−03
GO:0036293	response to decreased	*CA9, HIG2, EGLN3*,	8.79E−07	4.68E−03
	oxygen levels	*STC2, ANGPTL4*,		
		*CXCR4*		
GO:0070482	response to oxygen	*CA9, HIG2, EGLN3*,	1.29E−06	4.12E−03
	levels	*STC2, ANGPTL4*,		
		*CXCR4*		
GO:0036294	cellular response to	*CA9, HIG2, EGLN3*,	4.02E−05	4.94E−02
	decreased oxygen levels	*STC2*		
GO:0070887	cellular response to	*CA9, HIG2, INHBB*,	2.17E−05	3.46E−02
	chemical stimulus	*EGLN3, STC2, MMP9*,		
		*CXCR4, CSF1R, FN1*,		
		*PFKP, CD36*		
GO:0042221	response to chemical	*CA9, HIG2, INHBB*,	3.00E−05	4.35E−02
		*EGLN3, STC2, MMP9*,		
		*CXCR4, CSF1R, FN1*,		
		*PFKP, SLC16A3*,		
		*C1QA, CD36*		
GO:0006954	inflammatory response	*TYROBP, CXCR4*,	6.62E−06	1.51E−02
		*CSF1R, FN1, C1QA*,		
		*CD36*		
	positive regulation of	*INHBB, EGLN3*,	2.88E−06	7.65E−03

The results from the Cox proportional hazards model are shown in **Table 5**. Our results showed that all seven studied genes showed a significant relationship (p = 0.001–0.047) with survival when using the Cox model.

**Table 5:** Association of gene expression with progression free survival in analysis using the Cox proportional hazards model.

Gene	Cox regression, p =
*CA9*	<0.001*
*NDUFA4L2*	0.002*
*EGLN3*	0.006*
*BHLHE41*	0.003*
*VWF*	0.003*
*IGFBP3*	0.047*
*ANGPTL4*	0.016*

*Significant with FDR correction for multiple comparisons.

**Figure 4:**
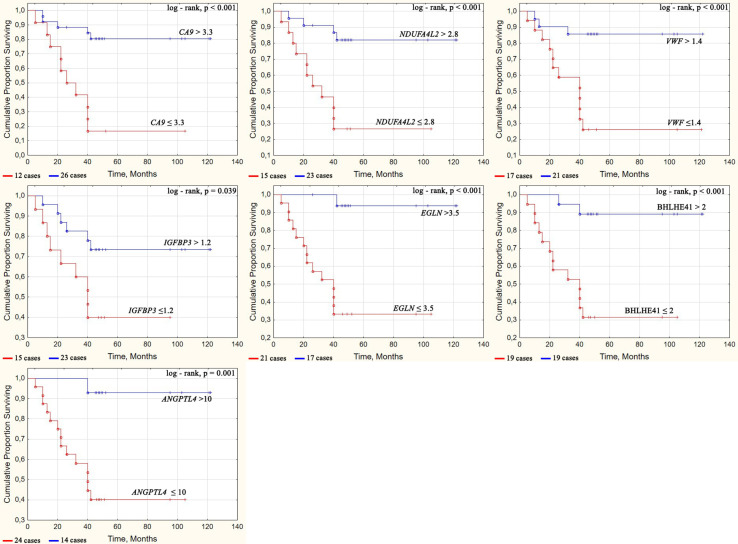
Survival curves of ccRCC patients depending on the gene expression level. Some cases have values that are close in time, so they may not be easily distinguishable on the graph. The number of cases for each Kaplan–Mayer curve is given under the graphs.

Next, we grouped patients based on their survival outcomes. The main survival parameters were determined using the Kaplan–Meier survival curve, as depicted in **Figure 4**. A tipping point in the curve can be observed at 42 months (3.5 years) as well as a separation of high and low mortality density.

Given these results, the patients were classified into two groups; i.e., those living for more than 42 months without progression of the disease (23 people) and those living less than this period from the moment of diagnosis (15 people). ROC analysis was used to analyze the relationship between gene expression levels and survival in these groups of patients (**Table 6**).

**Table 6:** Relationship between gene expression and survival by comparing groups of patients living less and more than 3.5 years using ROC analysis.

Gene	Area under ROC curve		95% CI	Cutoff value	Significance level P (Area = 0.5) (AUC)	Sensitivity	Specificity
*CA9*	0.8	20	0.662–0.926	≤3.3	<0.001*	66.7	91.3
*NDUFA4L2*	0.7	78	0.614–0.896	≤2.8	0.001*	73.3	82.6
*EGLN3*	0.8	59	0.708–0.950	≤3.5	< 0.001*	93.3	69.6
*BHLHE41*	0.8	46	0.693–0.942	≤2	<0.001*	86.7	73.9
*VWF*	0.8	35	0.679–0.935	≤1.4	<0.001*	80.0	78.3
*IGFBP3*	0.6	59	0.488–0.805	≤1.2	0.09	60.0	73.9
*ANGPTL4*	0.7	52	0.586–0.877	≤10	0.002*	93.3	56.5

*Significant with FDR correction for multiple comparisons.

The ROC analysis revealed that the expression of six of the seven analyzed genes (*CA9*, *NDUFA4L2*, *EGLN3*, *BHLHE41*, *VWF*, and *ANGPTL4*) showed a statistically significant relationship (p = 0.001–0.002) with the survival of patients with ccRCC. The differences remained significant when an FDR correction was applied, i.e., a decrease in the level of expression of *CA9*, *NDUFA4L2*, *EGLN3*, *BHLHE41*, *VWF*, and *ANGPTL4* is a factor in the unfavorable prognosis of ccRCC.

For the analysis of patient survival in each group and each gene, ROC analysis was used to select the cutoff values that showed the best sensitivity and specificity when using expression levels as a classifier (**Table 6**). The frequencies of expression above and below the threshold were determined in these two groups. The results are presented in **Table 7**. When analyzed using Fisher’s exact test, the presence of an association of expression levels of the all seven genes (p = 0.001–0.049) with a life span of more and less than 3.5 years, was found (**Table 7**).

**Table 7:** The frequencies of gene expression levels relative to the cutoff value among ccRCC patients living more and less than 3.5 years and characteristics of the association of gene expression with the life duration.

Gene	Frequency of expression higher/lower from the cutoff value for those living over 3.5 years	Frequency of expression higher/lower from the cutoff value for those who have lived less than 3.5 years	Odds ratio/ 95 % CI	Relative risk/95%CI	Fisher exact two-tailed, p=
					
			23.00/	8.33/	
*CA9*	21/2	5/10			<0.001*
			3.80–139.15	2.10–33.01	
			13.06/	4.22/	
*NDUFA4L2*	19/4	4/11			<0.001*
			2.71–62.93	1.64–10.81	
			32.00/	3.07/	
*EGLN3*	16/7	1/14			<0.001*
			3.49–293.08	1.63–5.77	
			18.42/	3.32/	
*BHLHE41*	17/6	2/13			<0.001*
			3.18–106.59	1.62–6.80	
			14.40/	3.68/	
*VWF*	18/5	3/12			<0.001*
			2.89–71.82	1.63–8.32	
			4.25/	2.3/	
*IGFBP3*	17/6	6/9			0.049*
			1.06–17.07	1.03–5.13	
			18.20/	2.15/	
*ANGPTL4*	13/10	1/14			0.002*
			2.04–162.60	1.32–3.49	

*Significant with FDR correction for multiple comparisons.

The cutoff values were used to analyze the relationship between the mRNA expression and ccRCC survival by assessing the significance of the differences in the survival curves according to the Kaplan–Meier analysis.

Survival curves were constructed by classifying patients based on the level of gene expression that was above or below the cutoff value as determined by ROC analysis (**Table 6**). Significantly different (p = 0.001–0.039) survival curves were assessed using the log-rank test depending on *CA9, NDUFA4L2, EGLN3, BHLHE41, VWF, IGFBP3*, and *ANGPTL4* expression as shown in **Figure 4**.

The overall survival rates were significantly higher in patients who had tumors with increased expression of *CA9*, *NDUFA4L2*, *EGLN3*, *BHLHE41*, *VWF*, *IGFBP3*, and *ANGPTL4* compared with patients who had reduced expression for these genes. On average, the proportion of patients that survived for more than 3.5 years was five times different at high and low levels of these gene expression.

Consequently, the genes *CA9*, *NDUFA4L2*, *EGLN3*, *BHLHE41*, *VWF*, and *ANGPTL4* had a significant association with survival as demonstrated by four statistical methods. *IGFBP3* had a significant association with survival in only three of the four statistical criteria used, and therefore, the reliability of its association with survival was lower.

The high transcriptional activity of the genes *CA9*, *NDUFA4L2*, *EGLN3*, *BHLHE41*, *ANGPTL4*, *VWF*, and *IGFBP3* was associated with a favorable prognosis for patient survival, whereas low activity was associated with unfavorable survivability. Additionally, four genes, i.e., *CA9*, *EGLN3*, *BHLHE41*, and *VWF*, had an AUC >0.8 based on the ROC analysis, indicating that their expression may serve as a very good classifier for the life expectancy of patients with ccRCC.

To increase the diagnostic ability of the identified genes, a panel of several genes was used as a marker for predicting the 3.5-year survival as an age that separate of high and low mortality density. The panel was based on the presence/absence of the combined increase in the expression of several genes in each sample. Using this panel of markers, we obtained a test with sufficiently high characteristics (**Table 8**).

**Table 8:** Details of the panel of genes—candidates prognostic markers longevity upon expression analysis of several genes.

Gene group	Sensitivity/Specificity	Probability to live more than 3.5 years/95% CI	Odds ratio/95% CI	Relative risk/95 %CI
Two genes of *VWF*				
*BHLHE41*	100%/82.61%	100%	134.33/	5.75/
*EGLN3*			6.71–2,689.95	2.36–14.01
Three genes of	80.00%/91.30%	87.50%/		
*CA9 VWF*		71.63–95.10%	42.00/	9.20/
*BHLHE41 EGLN3*			6.13–287.82	2.39–35.43

Specifically, high sensitivity and specificity were obtained when classifying patients on survival on the base of the combined expression levels of several genes. Using a panel of three genes, i.e., *VWF*, *BHLHE41*, and *EGLN3*, with any two genes below the threshold value considered a marker, it was theoretically possible to predict survival with 100% probability, high OR, and RR values. However, in practice, such a panel may be insufficient due to experimental variations. A panel that similarly uses four genes as markers for ccRCC may be more reliable. Although the sensitivity values for the application of this method were lower, the risk characteristic (RR = 9.2) was high. For the prediction of individual patient survival, we used the following formula: if the expression level (in a given ccRCC sample) of three or four of these genes was reduced, the resulting lifespan is less than 3.5 years; conversely, if it was increased, the lifespan is longer than 3.5 years. If the number of genes showing increased and decreased expression is equal, the result is undefined. The simultaneous analysis of the expression of the *VWF, BHLHE41*, and *EGLN3* genes to determine prognosis will enable 89.47% of individuals to correctly predict a life expectancy of more than 3.5 years. The share of false positive results was 10.53%.

## Discussion

According to Gene Ontology analysis (GO), the main functional processes showing enrichment for the studied genes include the response to stress, hypoxia, chemical stimuli, as well as the activation of the metabolic and inflammatory processes, and the positive regulation of the multicellular organismal process. The genes *HIG2*, *INHBB*, *TYROBP*, *STC2*, *CXCR4*, *NNMT*, *FN1*, *PFKP*, *SLC16A3*, *C1QA*, and *CD36* did not show a significant change in expression during tumor progression.

Among the genes studied, *CA9*, *NDUFA4L2*, *EGLN3*, *VWF*, *IGFBP3*, *ANGPTL4*, and *BHLHE41* decreased their expression with the development of ccRCC and a significant association between their expression level and the TNM stage was demonstrated. The activation of these genes was observed at the initial stages of ccRCC development and was associated with GO processes such as the response to stress and hypoxia, mainly for the *CA9*, *EGLN3*, and *ANGPTL4* genes. Six of these genes, i.e., *CA9*, *NDUFA4L2*, *IGFBP3*, *BHLHE41*, *ANGPTL4*, and *EGLN3* are direct targets of the HIF-1 transcription factor (17–22). HIF-1 is a hypoxia-induced factor that under hypoxic conditions mediates the transcriptional activation of target genes by binding to the hypoxia response element (HRE) located in their regulatory regions. The activation of HIF-1 in the kidney is dependent on the prevalence of hypoxia as well as the presence of the VHL gene product (pVHL). pVHL is part of the E3 ubiquitin ligase complex, which under normal oxygenation conditions promotes the attachment of ubiquitin to hydroxylated HIF transcription factors, promoting their destruction *via* the proteasome pathway. Under hypoxic conditions or in the absence of a functional pVHL, the VHL complex does not bind to non-hydroxylated HIF transcription factors, leading to their accumulation in cells. HIF-1 causes overexpression of several genes (23). The activation of those genes with increased expression in the early stages of tumor development is associated with HIF-1-mediated cellular adaptation to the oxygen-deprived microenvironment as well as due to the accumulation of HIF1α caused by the inactivation of the VHL gene.

CA9 (carbonic anhydrase type 9) is a transmembrane glycoprotein. Its main function relates to the regulation of the pH and maintaining the acid-base balance. Its expression is associated with the von Hippel-Lindau protein (pVHL) and is regulated by the transcription factor HIF-1α (24, 25). In the early stages of tumor development, increased expression of CA9 is required as an adaptation factor for hypoxia. Due to glycolysis and mitochondrial respiration, lactate, CO2, and H+ are formed, acidifying the intracellular and extracellular environment. CA9 catalyzes the reversible reaction of H+ and HCO3- from H2O and CO2. Together with the NBC transporter complex, CA9 promotes the transfer of HCO3- and Na+ from the extracellular environment into the cell. Subsequently, Na+ enters the blood, where in the form of sodium bicarbonate it preserves the alkaline reserve of the blood. In combination with the monocarboxylate transporter (MCT), CA9 functions as a “proton antenna,” which ensures the rapid exchange of H+ and the transfer of lactate from the cell to the extracellular space. The kidneys are unique, in contrast to other organs, in that it excretes the excess of H+ protons from the extracellular environment into the urine, thereby preventing the acidification of the extracellular space (26, 27).

EGLN3 (PHD3) expression is also HIF-1α dependent (22). PHD3 is involved in the regulation of glucose metabolism. It regulates key glycolytic enzymes, including PFKP, TPI1, ENO1, PGAM1, and LDHA, along with the glucose transporter GLUT1. LDHA converts pyruvate to lactate. Thus, PHD3 maintains a high rate of glycolysis and lactate production in ccRCC tumors (28). Following from our data on the analysis of correlations, the *CA9* and *EGLN3* genes were coordinated and significantly increase their expression at the initial stage of ccRCC development. This may occur not only to regulation by the same transcription factor but also because the two genes function in the same metabolic chain. Moreover, a simultaneous increase in the expression of *CA9* and *EGLN3* was observed in the study for the functional relationship between gene transcriptional activity and the response to lactic acidosis and hypoxia in breast tumor cells (29).

Our results show that the expression of *EGLN3* was highly correlated with the expression of *NDUFA4L2*. *NDUFA4L2* encodes the NADH dehydrogenase subunit, which is a component of the mitochondrial respiratory chain. The physiological role of NDUFA4L2 is likely the fine regulation of oxidative phosphorylation through interaction with other subunits within complex 1 of the mitochondrial respiratory chain. *NDUFA4L2* is a direct target of the HIF-1 transcription factor, and its expression is activated under hypoxic conditions (18). Hypoxia-induced NDUFA4L2 attenuates mitochondrial oxygen consumption by inhibiting complex I activity, which limits intracellular ROS production under low oxygen conditions (30). The significance of NDUFA4L2 expression in the development of ccRCC was first demonstrated by research from our group (31) and Fu et al. (32) and was subsequently followed by the works of other researchers (12, 33–35). NDUFA4L2 expression is also increased in non-small cell lung cancer (NSCLC) cell lines cultured under hypoxic conditions. Additionally, HIF1α-activated NDUFA4L2 inhibits the production of ROS by the mitochondrial respiratory chain in NSCLC cells. NDUFA4L2 knockdown increased ROS production in NSCLC cell lines (36). Consequently, the high correlation between the expression of *NDUFA4L2* and *EGLN3* is due to their similar HIF-1-induced regulatory responses, and the functional combination of glycolysis activation with the decrease in mitochondrial oxygen consumption.

Interestingly, there was also a high correlation between *IGFBP3* expression and the *EGLN3* and *NDUFA4L2* genes. This may indicate a certain combination of the functions of these genes. IGFBP3 is a multifunctional protein that modulates the activity of the IGF/IGF-IR system, which plays different roles in blood circulation, in the extracellular environment, and inside the cell (37). *IGFBP3*, like *EGLN3* and *NDUFA4L2*, is a direct target of HIF-1 (19). IGFBP3 also performs many functions independent of the IGF system. In particular, it can participate in glucose metabolism, which is reflected in the enriched GO- processes for regulation of glucose metabolic process (https://www.uniprot.org/uniprot/P17936).

High expression of IGFBP3 leads to increased glucose levels (38) and increasing of glucose assimilation (39, 40). Our data on the co-expression of *IGFBP3* with *EGLN3* and *NDUFA4L2* support the idea that it participates in glucose metabolism in ccRCC.

ANGPTL4 is an angiopoietin-like protein 4 expressed by tumor and endothelial cells. Its expression is induced under hypoxic conditions and is regulated by several transcription factors, including HIF-1 and PPARs (21). Its most well known functions are its participation in lipid regulation and glucose metabolism. In addition, ANGPTL4 is involved in resistance to anoikis and changes to redox regulation. Its role in cancer development is controversial; it can both enhance and block angiogenesis and proliferation (41). In this study, the highest expression of *ANGPTL4* occurred at the initial stage of ccRCC development. Its activation in the tumor at this stage correlates with the expression levels of the other HIF-1-induced genes studied, i.e., *CA9*, *NDUFA4L2*, *EGLN3*, and *IGFBP3*. Such coordination of gene expression indicates that their functions are associated with the processes of energy metabolism, that is connected to the above-mentioned participation of *ANGPTL4* in glucose metabolism. Moreover, among the known functions of *ANGPTL4*, the task of adapting to the hypoxic conditions of stage I/II ccRCC tumor development may correspond to the prevention of cancer cell apoptosis and a shift in redox regulation towards ROS formation, which stimulates tumor progression (42, 43). This role corresponds to *ANGPTL4* participation in the GO processes we identified that related to cell death regulation and the positive regulation of multicellular organismal processes.

At the initial stage of tumor development, *BHLHE41* expression correlated with that of *VWF* and *IGFBP3* expression. BHLHE41 (DEC2) is a (basic helix-loop-helix (bHLH)) transcription factor, whose expression is activated under hypoxic conditions and regulated by HIF-1 (20). High levels of BHLHE41 have been identified in ccRCC in TCGA studies. At the same time, no connection between the expression of BHLHE41 and the classical adverse pathological factors and decreased survival were found (44). In another study, the increased expression of *BHLHE41* stimulated the proliferation of cultured ccRCC cells, and its knockdown led to a significant decrease in these indicators (45). Transfection of BHLHE41 into cultured cancer cells increased their proportion in the S and G2 phases and decreased them in the G1 phase (46). Therefore, increased BHLHE41 expression leads to an increase in the proliferative properties of tumor cells at the initial stage of ccRCC development. The *IGFBP3* gene, like *BHLHE41*, is a direct target of HIF-1α (19), which may be associated with its activation and co-expression with *BHLHE41*. It is also impossible to exclude, along with the possible participation in glucose metabolism described above, the influence of *IGFBP3* on proliferation. The known action of *IGFBP3* on cell proliferation is dual; it can both stimulate and block cell growth, which depends on its effect on the so- called phospholipid rheostat (47). In addition, *IGFBP3* stimulates the synthesis of sphingosine-1-phosphate (S1P), which activates proliferation (48). The *IGFBP3* in the composition of other genes was activated at the initial stage of ccRCC, and per GO processes, it participates in the metabolism of proteins, including lipoproteins. In the human renal cell carcinoma cell line Caci-2 that was derived from a primary tumor, IGFBP3 stimulates proliferation (49). This may be evidence that *IGFBP3* stimulates proliferation in the early stages of ccRCC. The fact that *IGFBP3* is involved in the same process as *BHLHE41* may be an additional reason that the genes are co-expressed. von Willebrand factor is a plasma protein synthesized by vascular endothelial cells and plays an important role in hemostasis. It mediates platelet adhesion and aggregation to damaged vessel walls by binding to other proteins, for example, coagulation factor VIII or collagen (type 1 alpha 1) (50). An increased VWF level observed in the clinical setting is an indicator of endothelial damage and is often considered as a factor contributing to tumor progression (51, 52). Although *VWF* is not a direct target of the HIF-1 transcription factor, a coordinated increase in its expression with genes activated by this factor may be due to an increase in glycolysis-related sodium concentration caused by the action of *CA9*. This increases sodium concentration in the blood, as discussed above. Increasing the sodium concentration results in VWF expression (53). Through VWF, tumor-activated platelets are delivered to the vessel wall. Activated platelets stimulate tumor growth and angiogenesis (54). As demonstrated recently, VWF can bind growth factors, including VEGF-A, platelet-derived growth factor-BB (PDGF-BB), and the fibroblast growth factor-2 (FGF-2), and function as a reservoir of growth factors, accelerating proliferation and angiogenesis (55).

Thus, the coordinated increase in the expression of the seven genes under investigation at the initial stage of ccRCC development determines the number of processes that take place during the adaptation of the tumor to hypoxia and its development under these conditions. These processes include the transition to glycolysis and the corresponding decrease in oxygen consumption by mitochondria (*EGLN3*, *NDUFA4L2*, and, apparently, *IGFBP3*); the maintenance of acid-base balance (CA9), shifted due to glycolysis; stimulation of tumor growth; and prevention of apoptotic cell death in difficult adaptation conditions (*BHLHE41*, *VWF*, and possibly *IGFBP3*, and *ANGPTL4*).

The analysis of GO processes associated with the genes whose levels of expression are not altered during tumor progression (*HIG2*, *INHBB*, *TYROBP*, *STC2*, *CXCR4*, *NNMT*, *FN1*, *PFKP*, *SLC16A3*, *C1QA*, and *CD36*) indicates that these genes are activated in the initial phase, and maintained at all stages of development of ccRCC metabolic and inflammatory processes and positive regulation of the multicellular organismal process. These functional features of ccRCC have been noted in previous studies (56–58); however, we identified their early onset and its preservation at all stages of tumor development, which was not previously described.

As shown by the coordinated decrease in the expression of the seven genes upon tumor development, the initial processes controlled by them are attenuated. This may be due to angiogenesis and a decrease in glycolysis along with an increase in the level of mitochondrial metabolism (59). These processes correspond to a decrease in the expression of *EGLN3* and *NDUFA4L2*. A decrease in *EGLN3* expression leads to a decrease in the level of glycolytic processes (28) and *NDUFA4L2*, leading to an increase in mitochondrial oxygen consumption (36). As the tumor develops, its size also increases, which can lead to difficulty in removing H+, the formation of which is catalyzed by CA9 from the tumor space and the tumor microenvironment where it accumulates. These circumstances lead to the emergence of acidosis. Although CA9 expression is reduced as the tumor progresses through the stages of ccRCC, even at stage IV its levels of expression never fall below those observed in normal tissues. The expression of *ANGPTL4* correlates with *CA9*, *NDUFA4L2*, *EGLN3*, and *IGFBP3*. These genes, as described above, are associated with ccRCC energy metabolism. Additionally, *ANGPTL4* is involved in glucose metabolism (60–62). A decrease in its expression is associated with a decrease in glycolysis in cancer cells and an increase in mitochondrial metabolism, which promotes metastasis (60). It can be assumed that participation in similar functional processes is the reason for the correlation between the expressions of these four genes.

Although increased expression of *BHLHE41* stimulates the proliferation of tumor cells, which is especially important for tumor growth at the initial stage of development, it also inhibits its further development. In the further stages, cell invasion and EMT are inhibited by *BHLHE41* (63), lending to this gene’s classification as a tumor suppressor (64). Suppression of EMT and metastasis by BHLHE41 has been observed in breast (65–67), endometrial, and pancreatic cancer (63, 68). A negative correlation between BHLHE41 expression level and tumor invasion, metastases, and TNM stage has been found in gastric cancer (69). Our results indicate a similar process occurs in ccRCC tumors.

Our results show that in ccRCC tumors, the initial increase in expression of *BHLHE41* decreases as the tumor stage progresses, which meets the needs of ccRCC development i.e., the nature of its effect on the tumor changes as it develops. In the stages after initial growth, the downregulation of *BHLHE41* helps to create conditions for EMT and the invasion of tumor cells. The expression of the *IGFBP3* gene is coordinated with *BHLHE41* as well as with other HIF-1α-activated genes, decreasing in expression as the tumor grows (**Figure 2**). The effect of *IGFBP3* expression on cellular processes depends on the interaction of its product with various surface and intracellular molecules, and specifically, on the activation of other functional systems (47). Consequently, the activity of *IGFBP3* can have different and even opposite effects on the development of different types of tumors. Its low expression can enhance tumor progression, or conversely weaken it, depending on the type of tumor (70). Our data supports the hypothesis that *IGFBP3* has different effects on tumors at different stages of ccRCC development. At the initial stage, in which the release of cells from the tumor for development is irrelevant, this gene can influence glycolytic processes and enhance proliferation. Further, as the spread of the tumor becomes more important, this is facilitated by a decrease in the expression of *IGFBP3* (70). This interpretation is consistent with the available information on the effect of IGFBP3 on ccRCC cultures: IGFBP3 blocked proliferation of a ccRCC metastatic tumor in culture and stimulated the proliferation of a primary tumor in culture (49). In murine xenografts, the inhibition of *IGFBP3* increased tumor growth (71).

A decrease in *VWF* expression coordinated with HIF-1α-regulated genes during tumor progression is apparently due to a decrease in *CA9* expression and a corresponding decrease in its catalytic function, which leads to a decrease in the level of Na+ in the blood, and accordingly, to a weakening of the stimulation of *VWF* expression. This leads to the elimination of the proapoptotic effect of *VWF* and promotes the proliferation of tumor cells (72, 73). Additionally, as per *in vitro* data, the inhibition of *VWF* expression in endothelial cells leads to an increase in their proliferation, migration, and tube formation, which correlates with angiogenesis (74).

Thus, the expression of the seven genes was simultaneously increased at the initial stage of ccRCC and was coordinately decreased during tumor progression. When these occurrences along with the functional properties of the genes are taken into account, it indicates the important role of this group of genes in the maintenance of cancer cell viability and the development of tumors. At the initial stage of tumor development, the activation of the genes under consideration provides adaptation to hypoxia, accompanied by a metabolic shift, i.e., the development of glycolysis and a decrease in oxidative phosphorylation (*EGLN3*, *NDUFA4L2*, *ANGPTL4*, *CA9*, and, apparently, *IGFBP3*). Tumor growth is promoted by activating proliferation (*BHLHE41*, *VWF*, *ANGPTL4*, and possibly *IGFBP3*), and preventing the apoptosis of cancer cells (*ANGPTL4*). After passing stage I/II, the expression of these genes begins to decrease. However, this does not imply a decrease in their role in the development of ccRCC. The downregulation of these genes promotes tumor metastasis by stimulating EMT and invasion of cancer cells (*BHLHE41*); the development of acidosis, which stimulates tumor cell migration (*CA9*); a decrease in glycolysis; and an increase in oxidative phosphorylation, which promotes metastasis due to a better energy supply (*EGLN3*, *NDUPFA4L2*, and *ANGPTL4*), accelerating the proliferation of metastatic tumor cells (*IGFBP3*), and improving angiogenesis (*VWF*).

These conclusions about the functional significance of these genes in tumor progression substantiate the association of a decrease in their expression with tumor-specific survival in ccRCC patients. The genes *CA9*, *EGLN3*, *BHLHE41*, and *VWF*, as determined by ROC analysis and AUC values, were very good classifiers of life expectancy in terms of expression level and are characterized by significant OR values between 14.4 and 32 and an RR between 3.07 and 8.33. This indicates their importance as potential biomarkers for predicting the survival of patients with ccRCC. Additionally, from a practical point of view, it is difficult to use the expression of individual genes as real prognostic markers due to experimental variations. It is possible to overcome this limitation by using a panel of several genes.

Although a panel of three genes showed the best values for sensitivity and specificity, a panel of four genes as indicated had the best experimental reliability.

Recently, several studies have been published aimed at identifying genes whose expression may be associated with the survival of patients with ccRCC (75–83). However, these studies were performed by analyzing the study of mRNA expression presented in publicly available databases and were mainly obtained using NGS, without verifying the results by more accurate methods on patient samples. The groups of genes identified in different studies whose expression is associated with survival differ significantly. The work (80) that was performed by analyzing microarray data gave similar results to ours and the authors identified an association with the survival of the *CA9* and *ANGPTL4* genes.

Based on the experimental analysis of patient samples *via* RT-PCR for preselected genes using bioinformatics analysis, we identified powerful classifiers of survival, formed a panel of genes, and demonstrated the possibility of determining the prognosis for individual patients.

## Conclusions

In this study, the expression levels and relationships between differentially expressed ccRCC genes in tumors at different stages of development were investigated,. We demonstrate for the first time that the expression of the genes *CA9*, *NDUFA4L2*, *EGLN3*, *BHLHE41*, *IGFBP3*, and *ANGPTL4*, regulated by HIF1, is largely correlated, and along with *VWF*, the expression levels of these genes during tumor progression decreased coordinately. The expression levels of other genes studied by us did not change after the initial stage of ccRCC development. These genes, as per the processes identified with GO, are associated with the activation of metabolic and inflammatory processes, indicating that these occur at an early stage of tumor development and persist throughout subsequent stages. Based on the known functions and characteristics of co-expression, the first group of genes is associated with the development of glycolysis and a decrease in mitochondrial metabolism during stage I/II of ccRCC (*EGLN3*, *NDUFA4L2*, and *CA9*), as well as with the activation of proliferation (*BHLHE41*, *IGFBP3*, *ANGPTL4*, and *VWF*). These results may indicate the importance of the coordinated regulation of glycolysis levels and mitochondrial metabolism, as opposed to the passive altering of the production of mitochondrial energy in response to oxygen availability. The relationship between *IGFBP3* expression and the highly coordinated gene expression for *EGLN3* and *NDUFA4L2* demonstrates the participation of these genes in energy metabolic processes. As the tumor progresses, a decrease in the expression of these genes can lead to a reversal in metabolic processes, i.e., a decrease in the level of glycolysis and an increase in oxidative phosphorylation. This increase in energy supply contributes to metastasis and is also facilitated by the stimulation of EMT, the invasion of cancer cells (*BHLHE41*), the development of acidosis (*CA9*), and an improvement in angiogenesis (*VWF*). These functional features connect these genes with the survival rate of patients with ccRCC. The panel of genes *CA9*, *EGLN3*, *BHLHE41*, and *VWF* predicted the survival for more than 3.5 years in patients with ccRCC, with a probability close to 90%.

The results obtained here revealed several new functional features of the studied genes and the associated mechanisms in ccRCC development, which may provide a direction for further experimental study. Verification on an additional specimen of ccRCC for practical application of the found survival markers is required.

## Data Availability Statement

The original contributions presented in the study are included in the article/**Supplementary Material**. Further inquiries can be directed to the corresponding author.

## Ethics Statement

The studies involving human participants were reviewed and approved by Ethics Committee of the Federal State Budgetary Institution “Research Centre for Medical Genetics”. The patients/participants provided their written informed consent to participate in this study.

## Author Contributions

AlK and VM contributed to the conception and design of the study. PA performed the formal analysis. NA performed the methodology. NA, PA, DM, and AnK performed the investigation. NA wrote the first draft of the manuscript. AK modified the discussion and edited the manuscript. PA and DM performed the visualization. AK performed the supervision. All authors contributed to the article and approved the submitted version. 

## Funding

The research was carried out within the state assignment and funding of the Ministry of Science and Higher Education of the Russian Federation.

## Conflict of Interest

The authors declare that the research was conducted in the absence of any commercial or financial relationships that could be construed as a potential conflict of interest.

The reviewer AL declared a shared affiliation with one of the authors, VM, to the handling editor at time of review.
